# Regulation of Transactivation at C-TAD Domain of HIF-1*α* by Factor-Inhibiting HIF-1*α* (FIH-1): A Potential Target for Therapeutic Intervention in Cancer

**DOI:** 10.1155/2022/2407223

**Published:** 2022-05-10

**Authors:** Soniya Rani, Subhadeep Roy, Manjari Singh, Gaurav Kaithwas

**Affiliations:** ^1^Department of Pharmaceutical Sciences, School of Biomedical and Pharmaceutical Sciences, Babasaheb Bhimrao Ambedkar University (a Central University), Vidya Vihar Raibereli Road, Lucknow 226025 (U.P.), India; ^2^Department of Pharmaceutical Sciences, Assam University, Silchar, Assam 788011, India

## Abstract

Hypoxia-inducible factor-1alpha (HIF-1*α*) is a major transcription factor that adapts to low oxygen homeostasis and regulates the expression of several hypoxic genes, which aid in cancer survival and development. It has recently piqued the interest of translational researchers in the disciplines of cancer sciences. Hypoxia triggers an ample adaptive mechanism mediated via the HIF-1*α* transcriptional domain. Anaerobic glycolysis, angiogenesis, metastasis, and mitophagy are adaptive mechanisms that support tumor survival by promoting oxygen supply and regulating oxygen demand in hypoxic tumor cells. Throughout this pathway, the factor-inhibiting HIF-1*α* is a negative regulator of HIF-1*α* leading to its hydroxylation at the C-TAD domain of HIF-1*α* under normoxia. Thus, hydroxylated HIF-1*α* is unable to proceed with the transcriptional events due to interference in binding of C-TAD and CBP/p300. From this review, we can hypothesize that remodeling of FIH-1 activity is a unique mechanism that decreases the transcriptional activity of HIF-1*α* and, as a result, all of its hypoxic consequences. Hence, this review manuscript details the depth of knowledge of FIH-1 on hypoxia-associated cellular and molecular events, a potential strategy for targeting hypoxia-induced malignancies.

## 1. Introduction

Cancer is a polygenic disease wherein cells start to grow continuously in an uncontrolled manner and form an abnormal mass of cells, known as a tumor. Tumors are further classified as benign or malignant. A benign tumor remains confined and affects only the originating part of the body. Malignant tumors are metastatic and spread to nearby organs through blood vessels and lymph streams. Malignant tumors become lethal due to their ability to migrate to nearby organs, causing mortality. Among all the established therapies for malignant tumors (surgery, chemotherapy, radiotherapy, immune therapy, and targeted therapy), the failure to recover solid tumors has been reported due to hypoxia and its consequences [1, 2]. Hypoxia is thought to be a defining feature of solid tumor growth. Several studies have reported that hypoxia plays a significant role in creating a hostile environment for the growth and survival of solid tumors via the stabilization of O_2_-regulated transcription factor hypoxia-inducible factor-1*α* (HIF-1*α*). In hypoxic cells, HIF-1*α* acts as a transcription factor that allows many adaptive/survival mechanisms to perform their essential biological functions in a different way than normal cells. Furthermore, hypoxic conditions have been linked to greater resistance to chemotherapy and radiotherapy, as well as a poor prognosis [3, 4].

In the last two decades, however, scientists have found it more difficult to modulate hypoxia signaling via HIF-1*α*. Prolyl hydroxylase-2 (PHD-2) and factor-inhibiting HIF-1*α* (FIH-1) are two oxoglutarate-dependent dioxygenases that regulate HIF-1*α* transcriptional activity. PHD-2 governs the activity of HIF-1*α* at a reasonably high intracellular concentration of O_2_ by proteasomal degradation of HIF-1*α*. A modest decrease in intracellular O_2_ concentration renders PHD-2 inactive, causing intracellular accumulation of HIF-1*α*. Nonetheless, the transcriptional activity of HIF-1*α* is regulated by FIH-1, even at low intracellular O_2_ concentrations [5]. Moreover, it has also been observed that even at ≥1% intracellular O_2_, FIH-1 regulates HIF-1*α* [6, 7]. However, transcriptional effects of HIF-1*α* have been reported in rapidly proliferating tumor cells even at >1% intracellular O_2_, suggesting the role of FIH-1.

In addition to HIF-1*α*, HIF-2*α* is another isoform of the alpha subunit of HIF, it is structurally related to HIF-1*α* (48% amino acids similar to HIF-1*α* and 83% similar to basic-helix-loop-helix domains), and the hydroxylation process is also similar but distinct to their biological functions within the human tissues [8]. Although HIF-1*α* has a wide range of action, the response to HIF-2*α* may be limited to being cell type-specific. During embryonic development, HIF-2*α* expression was assumed to be exclusive to vascular endothelial cells [9]. According to recent reports, HIF-2*α* is now found in cardiac myocytes, kidney fibroblasts, hepatocytes, pancreatic interstitial cells, intestinal epithelial cells, and lung type II pneumocytes [10]. However, the proportional relevance of these genes varies by cell type; for example, HIF-1*α* is mostly responsible for the response to hypoxia in the endothelium and breast cancer cells, but HIF-2*α* is responsible in renal carcinoma cells [11]. However, Hu and colleagues discovered for the first time that the HIF-1*α* is responsible for the control of glycolytic gene transcription and, as a result, that it stimulates the glycolysis pathway in cancer progression, whereas the HIF-2*α* is not responsible [12]. Not only this, Imamura and colleagues also explored the divergent role of HIF-1*α* and HIF-2*α* in SW480 colon cancer cells via the selective knockdown of the same isoforms. The HIF-1*α* knockdown cells caused lower rates of proliferation and migration; when these cells were transplanted into animals, they resulted in a 57% reduction in tumor size, not in the HIF-2*α* xenograft. Whereas the HIF-2*α* knockdown cells had no effect on cellular proliferation in SW480 cells, although colony formation in soft agar experiments was increased. Taking into consideration of the previous findings that HIF-1*α* is a vital element in tumor migration and invasion and much more important than HIF-2*α* in colon cancer [13]. A large number of evidences suggest that the alpha subunit of HIF-1 is the most prominent isoform which is linked with cancer development and progression [14–17]. Therefore, here, we describe the role of HIF-1*α* in regard to cancer progression.

In this review, we focus on the cellular responses to hypoxia by exploring transcriptional mechanisms of HIF-1*α*. Efforts have been made to explore the various transcriptional effects of HIF-1*α* regulated through intracellular O_2_ concentration and the role of PHD-2 and FIH-1. The authors have also tried to explore the possible reasons for transcriptional activation of HIF-1*α*, in the presence of FIH-1, through a reasoned hypothesis.

## 2. Hypoxia

Hypoxia is a condition in which there is a lack of O_2_ at the cellular level. In aerobic multicellular organisms, O_2_ works as a terminal electron acceptor, essential for the maintenance of cellular physiological activities including cellular respiration, energy production, and the synthesis of signaling molecules [18]. However, insufficient O_2_ supply can cause various diseases such as liver fibrosis, diabetes, atherosclerosis, dementia, cachexia, and cancer [2]. In O_2_-deprived conditions, cells actuate abnormally, resulting in rapid proliferation, requiring more O_2_. However, the body cannot meet the undesirably increased demand for O_2_, and the cells become hypoxic.

**Figure 1:**
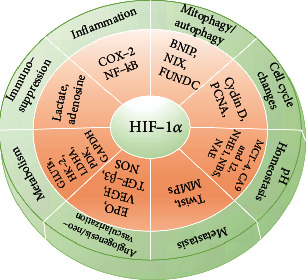
HIF-1*α*-associated genes and their role in hypoxia adaptive mechanisms. HIF-1*α* governs cancer physiology and associated genes. HIF-1*α*: hypoxia-inducible factor-1*α*; GLUTs: glucose transporters; HK-2: hexokinase-2; LDHA: lactate dehydrogenase A; PDK: pyruvate dehydrogenase kinase; GAPDH: glyceraldehyde 3-phosphate dehydrogenase; EPO: erythropoietin; VEGF: vascular endothelial growth factor; TGF-*β*3: transforming growth factor-*β*3; NOS: nitric oxide synthase; MMPs: matrix metalloproteinases; MCT-4: monocarboxylate transporter-4; CA 9 and 12: carbonic anhydrases 9 and 12; NHE1: sodium hydrogen exchanger 1; NBS: sodium bicarbonate symporter; NAE: sodium anion exchanger; BNIP3: B cell lymphoma 2 interacting protein 3; NIX: NIP3-like protein X; COX-2: cyclooxygenase-2; NF-*κ*B: nuclear factor kappa-light-chain-enhancer of activated B cells; PCNA: proliferating cell nuclear antigen.

Recent reports suggest that HIF-1*α* modulates genomic changes in cancer cells, which are responsible for angiogenesis, cell differentiation, anaerobic metabolism, pH homeostasis, mitophagy, and immunosuppressive pathways, all of which are required for cancerous cells to survive in O_2_-deprived conditions [9–12] (Figure 1).

Normal cells (≥21% O_2_) were situated close to the blood vessels. These cells efficiently use O_2_ to perform normal metabolic functions such as glycolysis, TCA cycle, and oxidative phosphorylation, which produce ATP as an energy source for survival and development. The cells situated far away from the blood vessels are known as moderately hypoxic cells (<21% to >1% O_2_) or OXPHOS cells. These cells perform the TCA cycle for energy production in comparison to normal and hypoxic cells. The more distantly located cells from the blood vessels which are highly deficient in O_2_ supply are called hypoxic cells (O_2_ tension < 1%). Due to insufficient O_2_ supply, these hypoxic cells repeatedly perform glycolysis to meet their energy requirements, and this phenomenon is termed as the Warburg effect. The Warburg effect was proposed in 19th century by Otto Warburg to understand the mechanism of metabolism in cancer cells [19]. According to Warburg, glycolysis is the predominant metabolic pathway of cancer cells for rapid energy production, compared to oxidative phosphorylation. Therefore, cancer cells undergo bypass catabolism of glucose into lactate through glycolysis, even under aerobic conditions. Under hypoxia, the glycolytic rate depends on glucose transporters (GLUTs) and the catalytic activity of glycolytic enzymes, namely, hexokinase-2 (HK-2), phosphofructokinase isoform L (PFK-L), and pyruvate kinase M2 (PKM2) [20]. Glucose transporters and enzymes are overexpressed during hypoxia, resulting in increased glycolysis and pyruvate accumulation. Accumulated pyruvate is further reduced to lactate through lactate dehydrogenase A (LDHA). To prevent intracellular acidosis, monocarboxylate transporter-4 (MCT-4) effluxes accumulated lactate to the extracellular matrix that is further used as fuel by nearby moderately hypoxic cells through monocarboxylate transporter-1 (MCT-1), wherein lactate is converted to pyruvate in the presence of lactate dehydrogenase B (LDHB). In moderately hypoxic cells, pyruvate is involved in the TCA cycle and produces ATP through oxidative phosphorylation for survival and development [14, 15].

### 2.1. Timeline of HIF-1*α* Transcriptional Development

In 1991, Semenza and coworkers discovered HIF-1 during their experiment on the hematopoietic gene erythropoietin. The hypoxia response element (HRE) regulates the hypoxic condition by the flanking region pentanucleotide sequence 5-RCGTG-3 on core DNA, which is named HRE [21]. Studies have revealed that transcriptional activation of hypoxic genes is initially regulated via binding of HRE to a specific protein that is stabilized under hypoxia, which was later identified as HIF-1 [22]. Further research found that HIF exists in three isoforms:HIF-1*α*, HIF-2*α*, and HIF-3*α*. HIF-1*α* and HIF-2*α* have 85% structural similarity to heterodimerization with the HIF-1*β* subunit [23]. HIF-3*α* differs from the other two isoforms in terms of structure and tissue specificity, implying that it has a different role [24].

**Figure 2:**
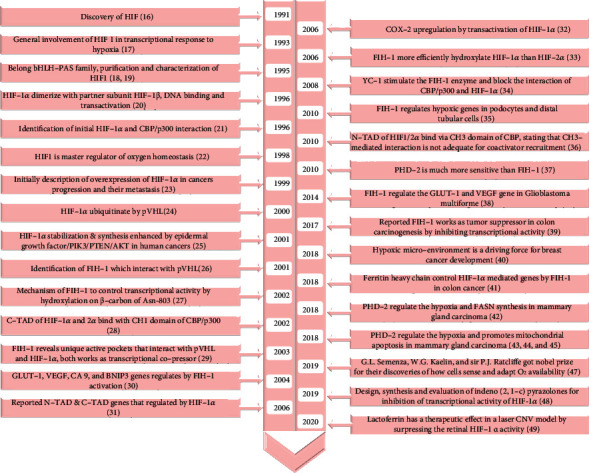
Timeline of HIF-1 represents the evolution and discovery of mechanistic intervention up to the years mentioned. HIF-1-related research is effective from the initial discovery of HIF-1 to work carried out recently in the adjunctive field. HIF-1: hypoxia-inducible factor-1; HIF-1*α*: hypoxia-inducible factor-1*α*; HIF-1*β*: hypoxia-inducible factor-1*β*; bHLH-PAS: basic helix-loop-helix/Per-ARNT-Sim; N-TAD: N-terminal transactivation domain; C-TAD: C-terminal transactivation domain; CBP/p300: cAMP response element-binding protein and p300 protein; GLUT-1: glucose transporter-1; VEGF: vascular endothelial growth factor; CA 9: carbonic anhydrase 9; BNIP3: B cell lymphoma 2 interacting protein 3; pVHL: von Hippel-Lindau protein; FIH-1: factor-inhibiting HIF-1*α*; COX-2: cyclooxygenase-2; PHD-2: prolyl hydroxylase-2; FASN: fatty acid synthase; CNV: choroidal neovascularization.

Numerous studies have been extensively made in terms of the structural, functional, physiological, and pathological aspects of HIF-1 [20, 25–27]. Clinical studies have revealed that HIF-1*α* plays a crucial role in cancer progression, metastasis, and poor prognosis. [28]. On October 7, 2019, three scientists, Drs. William G. Kaelin, Jr., Peter J. Ratcliffe, and Gregg L. Semenza, were awarded the Nobel Prize for their remarkable work on how cells sense and rapidly adapt to low O_2_ tension and provide a surface for understanding the mechanism by which life survives in a hypoxic environment. For a long time, the means of cell adaptation in O_2_-depriving conditions were unknown before these three Nobel Laureates discovered it [29]. This notable discovery provides new avenues for researchers to design novel strategies to counteract fatal diseases. A schematic representation of various reports on the chronology of HIF is outlined in Figure 2.

### 2.2. Structure and Molecular Mechanism of HIF Regulation

**Figure 3:**
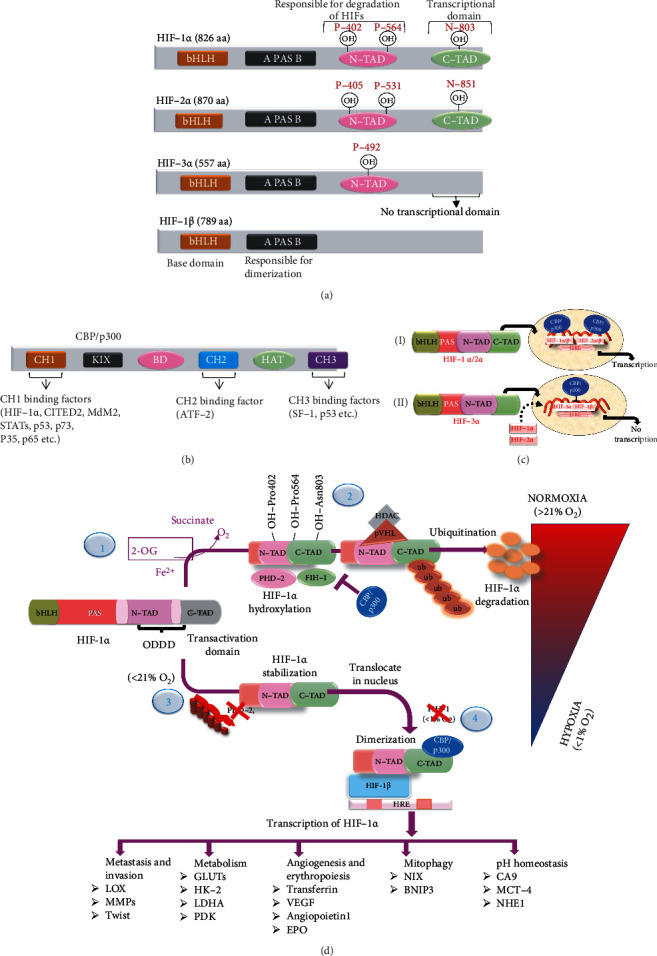
Structure of HIFs and CBP/p300 and their regulatory mechanism in normoxic and hypoxic conditions. Structural representation of different isoforms of HIFs and protein structure of CBP/p300 with different binding domains for transactivation. (a) HIFs show similarity in the base domain bHLH and PAS domains responsible for DNA binding and dimerization with homologous subunits. HIFs are divided into two subunits (*α* and *β*). The subunit is further divided into three isoforms (HIF-1*α*, HIF-2*α*, and HIF-3*α*). HIF-1*α* and -2*α* have 85% sequence similarity with the bHLH, N-TAD, and C-TAD domains. HIF-3*α* has no C-terminal domain (essential for transcription). (b) Representative band-like schematic diagram of the CBP/p300 proteins containing three coactivator domains, namely, CH1, CH2, and CH3, that play an important role in transactivation when binding with certain transcriptional factors. (c) Transcriptional mechanisms of HIFs and CBP/p300. (I) The C-TAD domain of HIF-1*α* and HIF-2*α* is responsible for binding with the transcriptional cofactor CBP/p300, thus increasing transcription of HIF-1*α* and HIF-2*α*. (II) HIF-3*α* does not contain the C-TAD domain; it is assumed that it is not involved in transcription. (d) Regulatory mechanism of HIF-1*α* by dioxygenase-dependent enzymes (PHD-2 and FIH-1) under normoxia and hypoxia, shown in red to blue color on the right side of the diagram. (1) Under normoxia (≥21% O_2_), HIF-1*α* is regulated by PHD-2 and FIH-1. PHD-2 is responsible for the hydroxylation of two proline residues (P-402 and P-564) of ODDD at N-TAD. At the same time, FIH-1 hydroxylates one asparagine (Asn-803) residue of C-TAD under normal conditions of O_2_, 2-OG, ascorbate, and Fe (II). (2) After that, the hydroxylated site of N-TAD is recognized by the pVHL protein, which functions as a tumor suppressor protein and binds with them for proteasomal degradation through ubiquitination by activation of E3-ubiquitin ligase. In contrast, hydroxylated C-TAD impedes the interaction of CBP/p300 proteins, thereby inhibiting the transcriptional program of HIF-1*α*. (3) When the O_2_ availability is low (<21% O_2_), PHD-2 becomes inactive, leading to the stabilization of HIF-1*α*. In comparison, FIH-1 regulates the discerning characteristic in low oxygen tension (≥1% O_2_) and regulates the transcriptional function of HIF-1*α*. (4) Under hypoxia (<1% O_2_), HIF-1*α* is fully stabilized and further processed for the transcriptional program after binding with CBP/p300 proteins because FIH-1 inactivates, and it is not able to interrupt the C-TAD and CBP/p300 complex. This complex further enters the nucleus, dimerizes with the partner subunit HIF-1*β*, and binds with the HRE protein, promoting cancer survival and individual gene expression. This complex then moves into the nucleus, dimerizes with its companion subunit HIF-1*β*, and binds to the HRE protein, expressing hypoxic genes that promote cancer progression.

HIFs are heterodimer proteins identified in Drosophila belonging to the basic helix-loop-helix/Per-ARNT-Sim (bHLH-PAS) family [30]. HIFs consist of O_2_-sensitive *α*- and O_2_-insensitive *β*-subunits within the NH2-terminal bHLH (responsible for DNA binding) and two PAS domains (responsible for dimerization of two subunits of HIFs). The subunit of HIF is found in three isoforms (HIF-1*α*, -2*α*, and -3*α*), which contain an oxygen-dependent degradation domain (ODDD). ODDD is responsible for proteasomal degradation via ubiquitination at the N-terminal transactivation domain (N-TAD). HIF-1*α* and HIF-2*α* have a C-terminal transactivation domain (C-TAD), while HIF-3*α* does not [21, 22] (Figure 3(a)). Multiple studies have reported that the C-terminal domain is essential for transcriptional functions [23, 31]. Studies have reported that HIF-3*α* is of pivotal importance in negatively regulating the transcriptional activity of HIF-1*α* and -2*α* [32, 33]. HIF-3*α* binds to the partner subunit HIF-1*β* to form a heterodimer complex. The complex binds to HRE by disrupting the binding of the HIF-1*α*/-2*α*–HIF-1*β* dimer complex, which is responsible for transcription (Figure 3(c)). The HIF-1*α* isoform is highly overexpressed in cancerous hypoxic cells, whereas rapid degradation is observed during normoxia (approximate *t*_1/2_ > 5 min). For activation of HIF-1*α* during hypoxia, sequential processes are involved, such as HIF-1*α* stabilization, nuclear translocation, heterodimerization, and transcriptional activation. The HIF dimer interacts with cofactor proteins [cAMP response element-binding protein and p300 protein (CBP and p300)]. It binds to HRE in the nucleus to increase the expression of myriad hypoxic transcriptional genes implicated in angiogenesis, cell proliferation, metastasis, pH homeostasis, hastening of the antitumor immune response, and resistance to chemotherapy [28, 34].

### 2.3. CBP and p300 Proteins

CBP and p300 are nuclear proteins that function as transcriptional coactivating proteins for various transcriptional factors including HIF-1*α* and STATs. These proteins are structurally homologous and work together to remodel the chromatin of DNA through histone acetyltransferases and therefore promote binding with promoter genes under hypoxia [35].

These proteins have three cysteine histidine-rich domains, namely, CH1, CH2, and CH3 that interact with and regulate the transcriptional activities of transcription factors (Figure 3(b)). The CH1 domain of CBP/p300 proteins facilitates scaffolding enveloped to the C-TAD domain of HIF-1*α*. It develops extensive hydrophobic and polar contacts, resulting in the formation of the CH1 and C-TAD complex that enhance cancer growth [29, 30, 34]. This complex is essential for the transcriptional activity of HIF-1*α*. The dissociation of this complex is associated with the attenuation of cancer growth. A study has reported that the interaction of the CH1 domain of CBP/p300 with C-TAD of HIF-1*α* is involved in mediating the expression of hypoxic angiogenic genes, including VEGF and EPO [36]. Recent studies have also revealed that the CH1 domain of CBP/p300 and the C-TAD of the HIF-1*α* complex is preferentially required for the expression of a multitude of hypoxic genes (GLUT-1, LDH-A, MCT-4, and VEGF) involved in cancer progression and adaptation to the hypoxic tumor microenvironment [24, 31, 36–38]. However, few studies have demonstrated that the CH3 domain of CBP/p300 interacts with N-TAD and activates HIF-1 transcriptional activity to a lesser extent [39]. Although many studies have reported the role of the CH1 domain in cancer progression, the role of CH2 and CH3 domains in gene transcription remains unexplored.

## 3. Regulation of HIF-1*α*

### 3.1. The Regulatory Mechanism of HIF-1*α* Stabilization by PHDs

PHDs are enzymes belonging to the O_2_, 2-oxoglutarate (2-OG), ascorbate, and Fe (II)-dependent family of dioxygenases that are responsible for proteasomal degradation of HIFs via the E3 ubiquitin ligase complex during normoxia. There are three isoforms of PHDs: PHD-1, PHD-2, and PHD-3 [23]. It has been reported by Pientka and colleagues that PHD-2 is primarily responsible for hydroxylation of HIF-1*α* in the nucleus. In this study, the authors created the mutant of PHD-2 that is limited to cytoplasm wherein it hydroxylates the HIF-1*α* to the lesser extent than the PHD-2 mutant present in the nucleus [40]. On the basis of the findings of this study, the authors would like to mention that PHD-2 nuclear localization plays a critical role in the regulation of HIF-1*α*, as well as the development and progression of cancer. PHD-1 is only found in the nucleus, whereas PHD-3 can be found in both the nucleus and the cytoplasm [41, 42]. PHD-1 hydroxylates two proline residues (P-405 and P-531) of HIF-2*α*; PHD-2 hydroxylates two proline residues (P-402 and P-564) of HIF-1*α* and PHD-3 hydroxylates at one proline residue (P-492) of HIF-3*α*. It has been reported that PHD-2 is the most prominent isoform that hydroxylates HIF-1*α* compared to HIF-2*α* and HIF-3*α*. PHD-1 and PHD-3 [37] most prominently hydroxylate HIF-2*α*. A decrease in PHD-2 activity increases the stabilization of HIF-1*α* in hypoxic cells [43]. HIF-1*α* regulation is essential for modulating transcriptional activity under normoxia and hypoxia. PHD-2 hydroxylates proline residues (P-402 and P-564) in the N-TAD domain. Subsequently, the von Hippel-Lindau protein (pVHL) recognizes the hydroxylation site on proline residues and promotes HIF-1*α* degradation via E3 ubiquitin ligase-mediated 26S proteasomal degradation [44]. However, under low O_2_ tension (<21% O_2_), PHD-2 fails to regulate proteasomal degradation of HIF-1*α*. A slight decrease in O_2_ concentration (<21%) renders PHD-2 ineffective, thereby precipitating intracellular accumulation of HIF-1*α*. It would be appropriate to mention that PHD-2 solely regulates the proteasomal degradation of HIF-1*α* by acting on N-TAD, whereas the transcriptional activity of HIF-1*α* is mediated through C-TAD (Figure 3(d)). As a result, the inhibition of O_2_-dependent proteasomal degradation of HIF-1*α* only participates in the accumulation of HIF-1*α*, rather than the activation of transcription genes associated with cellular proliferation, angiogenesis, and metastasis, which in turn are regulated through C-TAD. We would also like to note that recent reports from our laboratory have also reported that PHD-2 works as a tumor suppressor by downregulating the expression of HIF-1*α* to combat breast cancer in an animal model [45–52]. Singh and colleagues reported that exogenous chemical activation of PHD-2 downregulates HIF-1*α* along with FASN expression [53]. Subsequent studies by Roy et al. reported that *ω*-3 (ALA) and *ω*-6 (GLA) fatty acids downregulated the expression of HIF-1*α*, with a concomitant increase in PHD-2 expression. The anticancer potential of ALA and GLA was reported to be associated with mitochondrial apoptosis and was hypothesized to have a significant effect on cellular hypoxia. However, the impact of ALA and GLA on the transcriptional potential of HIF-1*α* (as regulated through C-TAD) remains unexplored [50, 51].

### 3.2. Transcriptional Regulatory Mechanism of HIF-1*α* by FIH-1

Besides PHD-2, FIH-1 is the second O_2_ sensor that regulates the transcriptional activity of HIF-1*α* and HIF-2*α*, by hydroxylating their asparagine (Asn-803) and (Asn-851) residues, respectively [49, 50, 54]. Hydroxylation of Asn-803 impeded the C-TAD of HIF-1*α* with CBP/p300 [55] (Figure 3(d)). It would be appropriate to note that PHD-2 and FIH-1 stabilize and negatively regulate the transcriptional activity of HIF-1*α* by interacting with their specific N-TAD and C-TAD domains, respectively.

Notably, PHD-2 acts at higher intracellular O_2_ concentrations (≥21% O_2_), whereas FIH-1 continues to regulate HIF-1*α* even at low intracellular O_2_ concentrations (≥1%) (Figure 3(d)) [56, 57]. A decrease in FIH-1 expression is linked with increased HIF-1*α* expression in hypoxia [58]. Additionally, FIH-1 appears to be a hypoxic gene suppressor in the kidney, acting through HIF-dependent and -independent mechanisms [59]. Chen et al. found that FIH-1 inhibits the interaction between C-TAD of HIF-1*α* and CBP/p300 proteins in human colorectal cancer by hydroxylating the Asn-803 residue [60]. Moreover, Jin and colleagues found that the ferritin heavy chain inhibits the interaction of p300 and HIF-1 by Asn-803 hydroxylation, which reduces the production of hypoxic genes such as VEGF, CA 9, and GLUT1 [61]. Studies have also reported that amphotericin, bortezomib, and YC-1 inhibit the expression of EPO, VEGF, and PGK1 by stimulating FIH-1 [6, 7, 43]. In U87 cells, Wang reported that FIH-1 inhibits HIF-mediated transcription of GLUT-1 and VEGF genes under normoxia and hypoxia [62]. FIH-1 is more effectively inhibiting the HIF-1*α* transcriptional activity, in contrast to other isoforms of HIFs [63].

Furthermore, studies have shown that FIH-1 can also regulate HIF-1*α* through distinct mechanisms. Since FIH-1 may bind to both HIF-1*α* and pVHL, a ternary complex comprising FIH-1, pVHL, and HIF-1*α* could be responsible for some of the FIH-1-mediated inhibitions of HIF-1*α*, resulting in increased HIF-1*α* ubiquitous degradation [64]. Instead, FIH-1 can also function as a corepressor by impeding histone deacetylases (HDACs) from binding to DNA, a primary mechanism that prevents transcription factors from binding to DNA [65]. Reports have also supported that downregulation of FIH-1 expression through small-interfering RNA stimulates hypoxic genes, whereas FIH-1 overexpression curtails the face of these genes [66, 67]. The accumulated data suggest that FIH-1 regulates HIF-1*α* by Asn-803 hydroxylation and could also regulate the pVHL-mediated ubiquitous degradation of HIF-1*α* in normoxia.

### 3.3. Comparative Efficacy of PHD-2 and FIH-1 to Regulate HIF-1*α*

HIF-1*α* has two major domains: N-TAD and C-TAD, which play an essential role in regulating the hypoxic response through PHD-2 and FIH-1 enzymes. Both PHD-2 and FIH-1 belong to the dioxygenase family, and their activity depends on O_2_, 2-OG, iron, and ascorbate. FIH-1 has shown a high affinity for O_2_ and iron compared to PHD-2 because PHD-2 has a narrow opening of the active site and binds loosely with cofactors [68]. In contrast, FIH-1 has broad, unique functional pockets in which O_2_ and cofactors are tightly bound [69]. This property of FIH-1 allows it to work efficiently even at low O_2_ tension (≥1%), whereas PHD-2 becomes inactive. Furthermore, FIH-1 requires only one O_2_ atom to hydroxylate a single residue of asparagine (Asn-803), wherein PHD-2 requires two O_2_ atoms to hydroxylate at two proline residues. Consequently, FIH-1 can mitigate the transcriptional activity of HIF-1*α* at low O_2_ tension (≥1%) in comparison to PHD-2 (≤21%). From the above explanation, it is clear that FIH-1 modulates HIF-1*α* transcriptional activity by holding C-TAD even under low O_2_ tension (≥1%).

Tarhonskaya and colleagues affirmed the above finding, who reported a significant difference between the Km and Ki values of PHDs and FIH-1. PHDs have a Km value that is slightly higher than the concentration of dissolved O_2_ in the air (200 *μ*M), with no significant difference in all three PHDs (Km values of PHD-1, PHD-2, and PHD-3 are 230 *μ*M, 250 *μ*M, and 230 *μ*M, respectively). FIH-1 has a Km value for O_2_, approximately 90 *μ*M, 2.5 times lower than for PHDs. Consequently, a slight change in O_2_ concentration renders PHDs inactive, whereas FIH-1 activity remains unaffected. The same results were observed with another cofactor, *α*-ketoglutarate. Alternatively, ascorbate had the opposite effect. The Km and Ki values of the enzymes and their ligands are shown in Table 1, and Table 2 shows the specific PHD-2 and FIH-1 inhibitors that may be useful in the treatment of ischemic stroke. Since the Km values of FIH-1 for O_2_ and *α*-ketoglutarate are lower than those of PHDs, PHDs are more sensitive than FIH-1. FIH-1 can withstand lower O_2_ concentrations than PHDs [70]. The higher the Km value, the lower is the affinity for the substrate.

Apart from O_2_, TCA cycle intermediates, including fumarate, succinate, and citrate, also inhibit FIH-1 and PHD-2 with varying Ki values. Studies have shown that fumarate (60 ± 20 *μ*M) and succinate (460 ± 70 *μ*M) competitively inhibit the PHD-2 with specific Ki values, whereas the Ki values in the case of FIH-1 were approximately increased by 166-folds (>10000 *μ*M). These metabolites play an important role in creating a favorable environment for cancer cell progression through the stabilization and accumulation of HIF-1*α*, which causes the cell to become hypoxic [71]. Citrate is a significant metabolite that is involved in catabolic and anabolic processes, such as oxidative phosphorylation in mitochondria and fatty acid synthesis in the cytosol. Some *in vitro* studies revealed that citrate is a potent inhibitor of FIH-1 with a Ki value of 110 *μ*M compared to PHD-2, with a Ki value of 1800 *μ*M. Therefore, citrate accumulates in the cell via several metabolic pathways (such as glutaminolysis and conversion from acetyl-CoA to citrate through citrate synthase), which inhibits FIH-1 activity and boosts HIF-1*α* activity.

Henceforth, we can easily presume that the transcriptional activity of HIF-1*α* is regulated through FIH-1, which continues to work even at low O_2_ tension (≥1% O_2_ level). However, transcriptional effects of HIF-1*α* have been reported in proliferating tumor cells (OXPHOS cells) even at >1% intracellular O_2_, contradicting the findings that FIH-1 regulates transcriptional activity even at low O_2_ (≥1% O_2_). Several reports have mentioned the above but failed to justify the same hypothesis [5, 39].

However, before getting into the plausible reasons behind that, we would like to briefly elaborate on the transcriptional activity of C-TAD/N-TAD domain(s) of HIF-1*α*.

## 4. Transactivation of HIF-1*α*-Mediated Gene Expression in Hypoxic Cancer

HIF-1*α* is a chief transcription factor that modulates low O_2_ and nutritional stress by modulating the O_2_ supply and utilization via the vascularization surrounding the hypoxic environment. It encodes the hypoxic genes essential for cancer progression and enhances the susceptibility of cancer cells to radiotherapy and chemotherapy. In metabolic, angiogenesis, pH homeostasis, and mitophagy programs, HIF-1*α* transcription upregulates all of these genes. As discussed in the upcoming text, these adaptive programs are required to survive and develop hypoxic cells. [52, 53].

### 4.1. Glucose Metabolism and Homeostasis

**Figure 4:**
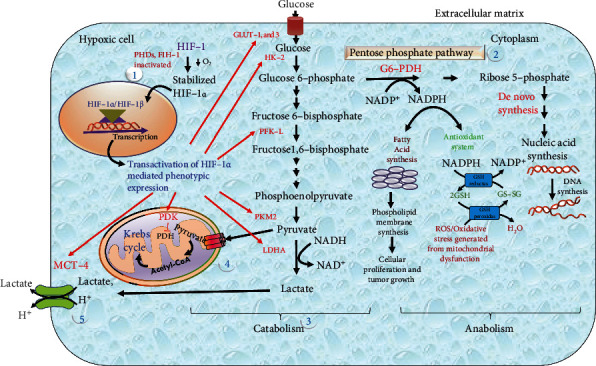
HIF-1*α* regulates metabolic reprogramming of hypoxic cells. Pictorial representation of glucose metabolism in hypoxic cells. (1) HIF-1*α* shows the mechanism of transcriptional activation under hypoxic conditions in which the degradation mechanism of HIF-1*α* is hindered by inactivation of oxygen-sensing enzymes (PHD-2 and FIH-1) at low oxygen tension, which leads to stabilization and transactivation of HIF-1*α*-mediated phenotypic expression of several enzymes involved in glycolysis. Hypoxic cells perform catabolism and anabolism with respect to HIF-1*α* activity. (2) The pentose phosphate pathway is an anabolic process for cancer cell survival and development. Conversion of glucose 6-phosphate to ribose 5-phosphate and NADPH in the presence of the G6-PDH enzyme involved in the biosynthesis (fatty acid synthesis and de novo synthesis) and antioxidant systems. It helps in building blocks and prevents oxidative stress in newly divided and proliferated cells. (3) Glucose is catabolized by a series of upregulated glycolytic enzymes, mainly HK-2, PFK-L, PKM2, and LDHA, which participate in the conversion of glucose into lactate and energy production in the form of ATP. (4) Mitochondrial bypass of pyruvate promoted the Warburg effect in hypoxic cells and expressed PDK, which inhibits pyruvate conversion to acetyl-CoA and avoids oxidative phosphorylation, which gives energy to normal cells. (5) MCT-4 is a membrane transporter that is strongly expressed in hypoxic cells and is involved in the efflux mechanism of glycolysis end products, lactate and H^+^, to avoid intracellular acidosis by effluxing lactate and H^+^ from the intracellular region to the extracellular matrix and subsequently promotes glycolysis for energy fulfillment.

Glucose is one of the most important requirements for cells to meet the energy requirements. In normal cells, glucose is mainly metabolized through glycolysis, TCA, and oxidative phosphorylation. The metabolism of glucose produces ATP, followed by the interconversion of isoenzymes [nicotinamide adenine dinucleotide hydrogen (NADH), flavin adenine dinucleotide (FAD), and adenosine diphosphate (ADP)], and an electron gradient in cells. Oxidative phosphorylation is the primary pathway of ATP production in normal cells. Under hypoxia, cells escape oxidative phosphorylation due to O_2_ deficiency and are forced to undergo anaerobic glycolysis, resulting in pyruvate accumulation. This pyruvate is further reduced to lactate by LDHA [72]. HIF-1*α* increases the flux of ATP via increasing the activity of rate-limiting glycolytic enzymes such as HK-2, PFK-L, and PKM2 (Figure 4). Increased glycolysis has been referred to as the Warburg phenomenon in hypoxic cells. Glucose transporters have also been found to be the first and most essential players in glucose influx, resulting in increased glycolysis in hypoxic cells [73]. Glucose not only participates in maintaining ATP requirements but also participates in the synthesis of nucleotides by upregulating the pentose phosphate pathway (PPP) in cancer cells. A study on breast cancer cells suggested that glucose consumption via PPP is enhanced in cancer cells [74].

In normoxia, FIH-1 regulates the impact of HIF-1*α* on the metabolic activity of the cells. During hypoxia, anaerobic glycolysis is regulated through HIF-1*α*-associated hypoxic genes. This effect has been shown in both *in vitro* and *in vivo* studies. The absence of FIH-1 boosted both oxidative phosphorylation and glycolysis in mouse embryonic fibroblasts [18]. One more similar study has supported that FIH-1 knockout mice have increased oxygen intake, hyperventilation, increased metabolic rate, decreased weight, and increased glucose and lipid homeostasis and are protected against weight gain and hepatic steatosis caused by a high-fat diet [75].

It is unclear whether the transcription of hypoxic genes is regulated through either N-TAD or C-TAD. A single study reported that glycolysis's GAPDH, PGK1, and PFK-L enzymes are regulated via N-TAD. In contrast, FIH-1 is not inhibited in moderate hypoxic cells; HK-2 and GLUT-1 genes are regulated via C-TAD [39]. However, it is challenging to determine which TAD controls the transcription of genes; therefore, there is a significant scientific gap that needs to be explored by researchers.

### 4.2. Intra- and Extracellular Acidity and pH Homeostasis

pH is essential and regulates the osmolality of intracellular and extracellular tissues, the rate of catalytic reactions, and the flow of ions. The pH creates a feasible environment for various catalytic enzymes to perform their physiological functions. During hypoxia, cells use a very high amount of glucose to produce ATP anaerobically. Consequently, pyruvate increases, which is further metabolized to lactate. Lactate accumulation increases intracellular acidity.

Consequently, cancer cells express a series of transporters [MCTs, sodium hydrogen exchanger 1 (NHE1), sodium bicarbonate symporter (NBS)] and enzymes [carbonic anhydrases 9 and 12 (CA 9 and 12)] to maintain pH homeostasis through mechanistic efflux of acidic metabolites to the extracellular environment [59, 60]. In particular, hypoxic cells exhibit an efflux mechanism against lactate accumulation and efflux of lactate from intracellular to extracellular space. Hypoxic cells express membrane transporter systems that function in favor of preventing acidosis [76]. MCT-4 is a highly expressed membrane transporter system in hypoxic cells. MCT-4 works to efflux lactate from the intracellular to the extracellular matrix [77]. Subsequently, these cells foster the rate of glycolysis in hypoxic cells and try to maintain a steady-state between the influx and efflux mechanisms. Extracellular lactate plays a pivotal role in a multidirectional manner by tracking hypoxic cells for proliferation, multiplication, and metastasis [78]. MCT-1 plays a vital role in the efflux mechanism of lactate by OXPHOS cells situated in a hypoxic environment. Therefore, extracellular lactate uptake by OXPHOS cells shows the complete metabolic TCA pathway for the maximum productivity of ATP. Moreover, lactate acts as an immunosurveillance agent against hypoxic cells [79].

**Figure 5:**
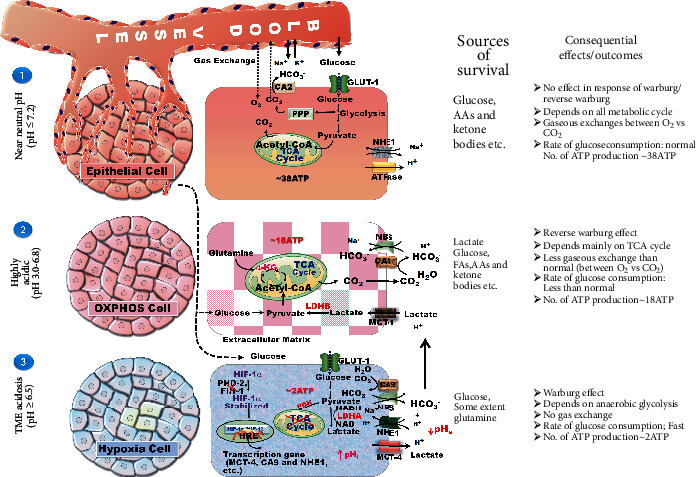
Effect of pH at different oxygen concentrations and depicting the source of survival and consequential outcomes in tumor progression. Effect of pH homeostasis within the cells (epithelial cells, OXPHOS cells, and hypoxic cells) and their coordination involved in the neutralization of the surrounding environment that are familiar to each other under different oxygen conditions. (1) In normoxia, cells maintain the intra- and extracellular effects of pH by efflux H^+^ ions through NHE1 and ATPase to the extracellular environment, and intracellular CO_2_ is neutralized by CA 2 in the form of bicarbonate and H^+^ ions, which are further directly exchanged by the Na^+^ K^+^ transporter that is situated on the blood vessels. Normal cells have a sufficient vasculature system for exchanging CO_2_ and O_2_ levels in the bloodstream. (2) OXPHOS cells are highly dependent on lactate as a source of energy compared to glucose and other supplements. In addition to lactate uptake, pH fluctuations may slightly decrease compared to hypoxic cells because lactate and other sources of energy may form the stagnant effect of their utilization by the OXPHOS cell. Lactate is transported by the MCT-1 transporter and converted into pyruvate. Glycolysis may produce some amount of pyruvate involved in ATP production (~18ATP) through the TCA cycle. Intracellular H^+^ ions are buffered through the CA 9 and NBS expression in the salt form of bicarbonates. (3) Cancer cells repeatedly rely on glycolysis for their energy demand (~2ATP) during hypoxia. Instead, pyruvate lactate accumulates in response to LDHA, thus increasing pHi. To avoid this acidic pHi, these cells upregulated the membrane transporter MCT-4, which effluxes intracellular lactate into the extracellular matrix. An increase in pHe is neutralized by other membrane transporters (NBS and NHE1) and CA 9 enzyme intracellularly and extracellularly, and extracellular lactate migrates towards OXPHOS cells, where it is consumed as an energy source.

Following the release of acidic metabolites into the extracellular environment, the extracellular pH (pHe) decreases. A decrease in extracellular pH supports tumor progression through distinct acid-sensing mechanisms, such as activating matrix metalloproteinases (MMPs) [80]. Lactate also promotes angiogenesis and antitumor immunity and is used by OXPHOS and tumor epithelial cells. The pH homeostasis in normoxic, OXPHOS, and hypoxic cells is shown in Figure 5. The literature has demonstrated that CA 9 and LDHA are regulated by C-TAD [24]. However, in the case of N-TAD, there is no available supporting literature. Therefore, it is worth noting that FIH-1 is an eminent negative regulator of pH-regulating genes through C-TAD of HIF-1*α* [39].

### 4.3. Angiogenesis and Neovascularization

**Figure 6:**
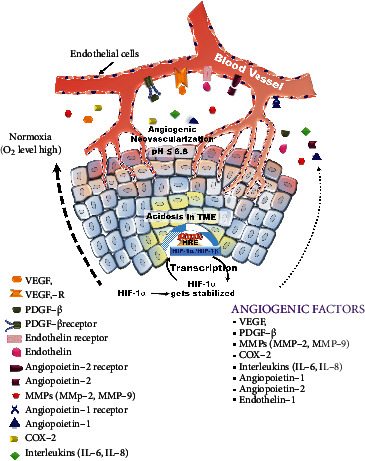
HIF-1*α* mediated angiogenic signaling in the tumor expansion. Angiogenesis is the formation of new blood vessels from preexisting blood vessels and plays a crucial role in the adaptation and migration of hypoxic tumors. Under hypoxia, various angiogenic factors are mentioned on the right side of the bottom, which is involved in angiogenesis. The core of the cancer masses causes an acidic TME due to a highly upregulated glycolysis-mediated end product, which leads to hypoxia where HIF-1*α* is stabilized and produces transcriptional activation of angiogenic genes. These angiogenic genes migrate extracellularly towards the blood vessel endothelium and bind with their respective receptors on the left side along with the respective agonist. This increases cell signaling towards remodeling and neovascularization of endothelial cells of the blood vessels. Neovascularization of blood vessels helps to provide supplements and maintain O_2_ homeostasis in hypoxic cells towards a normoxic effect. HIF-1*α*: hypoxia-inducible factor-1*α*; HIF-1*β*: hypoxia-inducible factor-1*β*; HRE: hypoxia response element; TME: tumor microenvironment; VEGF: vascular endothelial growth factor; VEGF: vascular endothelial growth factor receptor.

The growth of hypoxic tumors and angiogenesis is mainly dependent on the evolution of new blood vessels. Under hypoxia, HIF-1*α* is stabilized and expresses multiple genes to perpetuate angiogenesis. HIF-1*α* upregulates growth factors such as VEGF and primary fibroblast growth factor (bFGF) to promote vascular permeability, endothelial cell growth, and proliferation [81]. Moreover, FIH-1 reduces the expression of the angiogenic factor VEGF in head and neck carcinoma cells [82]. Other growth factors, such as platelet-derived growth factor-*β*, transforming growth factor (TGF), and angiopoietins, facilitate angiogenesis released by cancer cells. These growth factors play a crucial role in the vascularization and migration of cancer cells [83]. HIF-1*α* also promotes the expression of cyclooxygenase-2 and contributes to angiogenesis by promoting excessive secretion of several factors, particularly PGE2 [84]. It has been reported that HIF-1*α* encourages the expression of MMPs in cancer cells. MMPs are key players in the breakdown of the extracellular matrix (ECM) or baseline to stimulate endothelial cell metastasis and release associated growth factors that promote angiogenesis [80] (Figure 6).

HIF-1*α* promotes malignant characteristics and helps transfer tumor cells to distant organs with the subsequent expression of migrating genes and TGFs. Generally, cells become hypoxic because they exhaust the surrounding microenvironment, and there is no O_2_ and nutrition available for proper growth and survival. Therefore, these cells require migration to distant organs to fulfill their demand for O_2_ and nutrition. Studies have reported that tumor invasion and metastasis are dependent on the proteolytic modification of the ECM and loss of cell-cell adhesion [85]. Various genes such as Twist and Snail can promote angiogenic factors, including vimentin, cathepsin D, MMPs, keratin, and urokinase plasminogen activator (uPAR). Another important mechanism involved in invasion is the loss of E-cadherin expression by epithelial-to-mesenchymal transition (EMT). E-cadherin expression is controlled by Snail activation [5, 86]. These studies reported that FIH-1 regulates adrenomedullin, VEGF, and EPO angiogenic genes by acting on the C-TAD of HIF-1*α* [36, 39].

### 4.4. Mitophagy for Cell Survival Reprogramming

**Figure 7:**
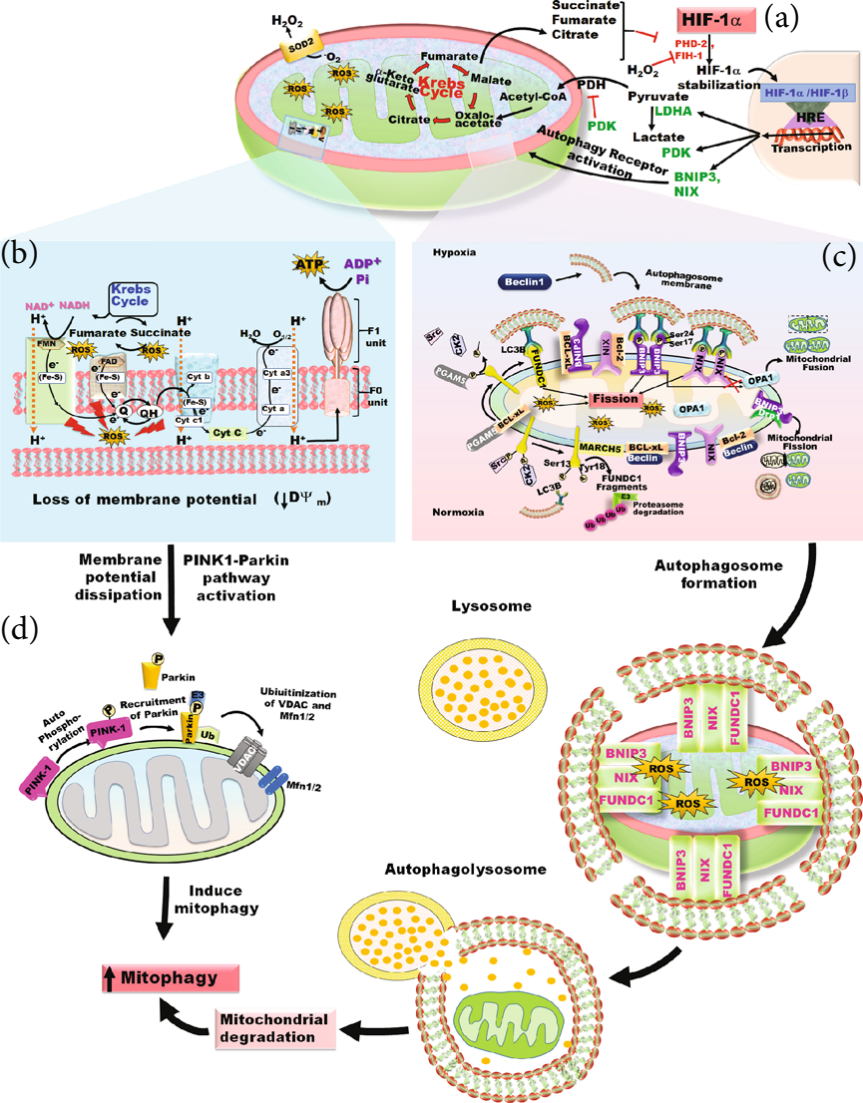
HIF-1*α* mediated induction of selective mitophagy under hypoxia. (a) Mechanism HIF-1*α* mediated upregulation of mitophagy response: HIF-1*α* gets stabilized and transacts various genes that regulate the cellular adaptive mechanisms under low oxygen tension under hypoxic stress. It promotes the glycolytic pathway for energy production and participates in the expression of PDK, which inhibits pyruvate entry into the TCA cycle. Hypoxia also disrupts MMP potential and produces ROS, which supports HIF-1*α* stabilization by inhibiting PHD-2 and FIH-1. TCA intermediates inhibit both enzymes due to competitive inhibition of their substrate. HIF upregulates the expression of the mitophagy receptors BNIP3 and NIX, which are essential in mitophagy induction. (b) Source of ROS generation in ETC: under hypoxia, insufficient O_2_ and accidental escape of electrons lead to ROS production. In anaerobic glycolysis, the NAD^+^/NADH ratio is altered in the cytoplasm, disturbing the MMP and translational modification of ETC components. ETC components, incredibly complexes I and III, cannot do the regular electron transportation and work as a host for ROS generation. Excessive ROS potentiates mitochondria's membrane integrity, leading to activation of the PINK-1/PARKIN pathway that involves ubiquitin-dependent mitophagy. (c) Role of BNIP3, BNIP3L/NIX, and FUNDC1 proteins in mitophagy: mitophagy receptors BNIP3, BNIP3L/NIX, and FUNDC1 are strongly expressed at the OMM of mitochondria in response to hypoxia. Under normoxia, CK2 and Src enzymes are responsible for phosphorylation at Ser13 and Tyr18 residues of FUNDC1, respectively. The phosphorylated FUNDC1 decreases the affinity towards binding with LC3B. The phosphorylated FUNDC1 is targeted for ubiquitination and subsequent degradation by mitochondrial E3 ubiquitin ligase by MARCH5. Under hypoxic conditions, dephosphorylation of FUNDC1 protein is carried out through the mitochondrial phosphatase PGAM5. The activity of PGAM5 is dynamically regulated by Bcl-2L1/BCL-xL protein through its BH3 domain. Bcl-2L1/BCL-xL proteins are situated on OMM during normoxia, but they do not interact with FUNDC1, although they do interact with PGAM5. After that, PGAM5 becomes inactive and inhibits dephosphorylation of FUNDC1, subsequently decreasing mitophagy. During hypoxia, PGAM5-Bcl-2L1/BCL-xL complex is dissociated, and Bcl-2L1/BCL-xL is degraded; a free form of PGAM5 interacted with FUNDC1 leads to dephosphorylation at Ser13, and Tyr18 residues of FUNDC1 subsequently interact with LC3B and trigger the mitochondrial autophagosome-mediated degradation. At the same time, BNIP3/NIX is upregulated and involved in the induction of mitophagy. Under normoxia, BECN1, which is linked to BCL-xL/Bcl-2 and the BNIP3/NIX protein, is still inactive. Under hypoxia, the BCL-xL/Bcl-2 proteins dissociate, allowing BECN1 to be released, which aids autophagosome formation and engulfs/envelops mitochondria for lysosomal degradation. BNIP3/NIX undergoes homodimerization and phosphorylation of Ser17 and Ser24 residues of proteins under hypoxia, and it interacts with LC3B, causing mitochondrial breakdown by increasing fission protein expression and decreasing OPA1 activity. *Fission and fusion process in mitophagy:* the fusion phenomenon also participates as the supplementary effect of mitophagy induction. The mitophagy receptors promote the fission process by upregulating the activity of the fission proteins Drp1 and Fis1. These proteins are also located on OMM and form the contact site for mitochondrial fragmentation. Under hypoxia, these proteins are upregulated in dysregulated mitochondria and in healthy mitochondria that induce mitochondrial segregation into the smaller fragment that is easily encapsulated by autophagosome for their degradation. In comparison, mitophagy receptors downregulate fusion activity under hypoxic response. The fusion protein, mainly OPA1, is involved in mitochondria's fusion activity, which is inhibited by mitophagy receptors. (d) The PINK-1/Parkin pathway for mitophagy induction: PINK-1/PARKIN pathway-induced mitophagy works in a ubiquitin-dependent manner. PINK-1 is transported into the IMM during normoxia, wherein it is cleaved by several proteases and finally degraded by ubiquitination. However, in hypoxia, dissipated MMP prevents entry of PINK-1 into the IMM, leading to accumulation of PINK-1 at OMM of mitochondria, wherein PINK-1 undergoes autophosphorylation which helps in phosphorylation as well as recruitment of PARKIN. The collection of PINK-1/PARKIN on the OMM triggers the E3 ubiquitin system which leads to mitochondrial autophagy.

Mitophagy is a program that eliminates dysfunctional/damaged mitochondria through phagocytosis. Mitophagy is one of the most critical mechanisms adopted by hypoxic cells to counteract increased ROS-mediated cell death [87]. In solid tumors, the mitochondrial-mediated production of ROS supplements tumor growth via HIF-1*α* signaling. ROS plays a crucial role in HIF-1*α* accumulation and subsequent stabilization by selective inhibition of PHDs and FIH-1 [88]. In contrast, in the hypoxic condition, mitochondrial-mediated production of excessive ROS causes more oxidative stress generated by intracellular H_2_O_2_ through the upregulated activity of superoxide dismutase 2. An excessive amount of H_2_O_2_ disturbs cell membrane integrity and promotes autophagy. Therefore, HIF-1*α* helps to cope with any damage to the mitochondria so that cancer cells evoke selective mitochondrial autophagy to maintain a healthy microenvironment (Figure 7(a)). HIF-1*α* regulates the mitochondrial mass directly and indirectly to overcome the oxidative stress-mediated impairment of cancer growth by activating ubiquitin-dependent and -independent pathways [72, 73].

#### 4.4.1. Ubiquitin-Dependent Mitophagy

In hypoxia, oxidative stress-induced mitophagy is executed through the PINK-1/PARKIN pathway. During anaerobic glycolysis, the NAD^+^/NADH ratio is altered in the mitochondrial matrix. After that, the ETC protein components (incredibly complexes I and III) cannot undergo everyday electron transportation and electron potential gradient across the membrane. After that, the altered concentration of substrates (succinate and fumarate, etc.) led to the accidental escape of electrons and ultimately decreased the mitochondrial membrane potential (MMP) (Figure 7(b)). During normoxia, PINK-1 is transported into the inner mitochondrial membrane (IMM) due to normal MMP, wherein it is cleaved by several proteases and finally degraded by ubiquitination. However, in hypoxia, the dissipated MMP prevents the entry of PINK-1 into IMM, leading to the accumulation of PINK-1 at the outer mitochondrial membrane (OMM). PINK-1 undergoes autophosphorylation and promotes the phosphorylation and recruitment of Parkin. The association of PINK-1/PARKIN on the mitochondrial surface triggers the E3 ubiquitin system and blocks voltage-dependent anion channel activity for supplemented effects. Thus, it leads to mitochondrial damage and selective mitophagy due to oxidative stress in a ubiquitin manner, as shown in [74, 75] (Figure 7(d)).

#### 4.4.2. Ubiquitin-Independent Mitophagy

Ubiquitin-independent mitophagy is another type of autophagy within cellular organelles that abrogates mitochondrial respiration and mass under hypoxia. FUN-14 domain-containing protein 1 (FUNDC1), BCL-2 adenovirus E1B 19 kDa-interacting protein 3 (BNIP3), and BNIP3-like/NIP3-like protein X (BNIP3L/NIX) are key mitophagy receptors responsible for the selective elimination of mitochondria in response to hypoxia [74, 89]. All these mitophagy receptors are found on OMM and similarly perform mitophagy by binding with their conserved LC3 interaction region (LIR) motif with light chain 3B (LC3B) site.

BNIP3 and NIX are proapoptotic proteins that participate in mitochondrial autophagy compared to total cell death, which is mediated by mitochondrial-mediated cytochrome c release and caspase activation. They work together to achieve selective mitophagy when mitochondria suffer from biological dysfunction, such as mitochondrial respiration (high ROS generation), MMP, and membrane permeability under hypoxia. Both BNIP3 and NIX contain one transmembrane domain with an N-terminus outside the mitochondria (cytoplasm), whereas the C-terminus is inside the mitochondrial intermembrane space (IMS). It has been reported that both genes are transcriptionally regulated via the involvement of CBP/p300 coactivators under hypoxic conditions. Interestingly, data revealed that the expression of BNIP3 is elevated in parallel with the transcriptional activities of HIF-1*α*, whereas nuclear factor kappa-light-chain-enhancer of activated B cells (NF-*κ*B) are responsible for its downregulation [90, 91].

Beclin 1 (BECN1) is an autophagy protein that participates in mitophagy through autophagosome formation. BECN1 is enveloped in the mitochondria and promotes lysosomal degradation under hypoxic stress. Bcl-xL/Bcl-2 proteins bind to BECN1 to form a complex (Bcl-xL-BECN1 and Bcl-2-BECN1), which negatively regulates the mitophagy response of BECN1. The complexes (BCL-XL-BECN1 and Bcl-2-BECN1) are dissociated by BNIP3 and NIX proteins during hypoxia, respectively, to release BECN1. BNIP3-BCL-XL and NIX-Bcl-2 complexes were homodimerized, followed by phosphorylation at Ser17 and Ser24 residues. Unbound BECN1 performs autophagosome formation followed by maturation, which promotes mitophagy during hypoxia [92].

Another mechanistic approach to hypoxia-induced mitophagy is the FUNDC1 receptor. Structurally, it contains three alpha-helices with a hydrophobic transmembrane domain. The N-terminus of the FUNDC1 receptor lies in cytosol, whereas the C-terminus lies in IMS. It has been reported that the cytosolic N-terminus of FUNDC1 interacts with light chain 3B (LC3B) and regulates hypoxia-induced selective mitophagy through reversible phosphorylation at Ser13 and Tyr18 residues [73, 74]. Under normoxia, casein kinase 2 (CK2) and tyrosine-protein kinase Src enzymes phosphorylate the Ser13 and Tyr18 residues of FUNDC1, respectively. After that, the stereochemical properties of FUNDC1 are altered, which decreases the affinity for binding with LC3B. Phosphorylated FUNDC1 is ubiquitinated and subsequently degraded by mitochondrial E3 ubiquitin ligase of the membrane-associated ring finger (C3HC4) 5 (MARCH5). Under hypoxia, Src and CK2 are inactivated and dissociated from FUNDC1. However, the mechanism underlying the inactivation of Src and CK2 remains unclear. The dephosphorylation of the FUNDC1 protein is carried forward through the mitochondrial phosphatase phosphoglycerate mutase family member 5 (PGAM5). PGAM5 is an enzyme similar to phosphoglycerate mutase (PGM) and is responsible for removing phosphate groups (dephosphorylation) in the metabolic cycles and producing metabolic disorders in mammals. The activity of PGAM5 is dynamically regulated by Bcl-2 family proteins, mainly Bcl-2L1/BCL-xL, through their BH3 domain. During normoxia, Bcl-2L1/BCL-xL is stationed on OMM with PGAM5 and is not associated with FUNDC1. The association of Bcl-2L1/BCL-xL with PGAM5 renders PGAM5 inactive, impeding the dephosphorylation of FUNDC1 and the subsequent inhibition of mitophagy. In contrast, the PGAM5-Bcl-2L1/BCL-xL complex is dissociated during hypoxia, and Bcl-2L1/BCL-xL is degraded. The free form of PGAM5 interacts with FUNDC1, leading to dephosphorylation of FUNDC1, followed by its interaction with LC3B to trigger mitophagy [93] (Figure 7(c)). Among the various genes associated with mitophagy, BNIP3 is regulated by N-TAD, whereas NIX (BNIP3L) is controlled via C-TAD of HIF-1*α*.

## 5. Clinical Studies to Targeting HIF-1*α* Signaling

Firstly, Zhong and colleagues provided clinical evidence indicating that HIF-1*α* plays a critical role in the growth and metastasis of several human cancers [28]. Various clinical studies have approved that HIF-1*α* is significantly linked to cancer progression, assisting tumor invasion, metastasis, angiogenesis, and the development of resistance to various available cancer therapies. Numerous drugs have been reported as HIF inhibitors and tested in clinical trials (Table 3). Many compounds have been found to be effective in the treatment of various types of solid tumors, including metastatic renal cell carcinoma, kidney cancer, ovarian cancer, and refractory solid tumors in clinical trials [94]. The HIF-1*α* signaling pathway is a potential therapeutic target for cancer treatment.

## 6. Conclusion and Future Perspective

As elaborated in the preceding paragraphs, the stabilization and transcriptional activation of HIF-1*α* are regulated through PHD-2 and FIH-1, respectively. However, one of the mechanisms is the intracellular O_2_ concentration, wherein the slight decrease in O_2_ concentration (<21% O_2_) renders PHD-2 inactive and prevents proteasomal degradation of HIF-1*α*. In solid tumors, this phenomenon can be easily observed in OXPHOS cells, leading to intracellular accumulation of HIF-1*α*. Irrespective of intracellular accumulation of HIF-1*α*, the transcriptional activity of HIF-1*α* is not anticipated, as the same is dependent upon the C-TAD and governed by FIH-1. It is appropriate to mention that FIH-1 is also an O_2_-dependent oxygenase. However, FIH-1 has a higher affinity towards HIF-1*α* and can even work at low intracellular O_2_ concentrations (≥1%). Overall, FIH-1 continues to regulate the HIF-1*α* transcriptional activity during normoxia, although the mechanism in OXPHOS and hypoxic cells is unknown.

The transcriptional effects of HIF-1*α* are evident in OXPHOS cells (>1% O_2_), which contradicts the finding that FIH-1 is active in OXPHOS cells and controls their transcriptional activity. Despite the availability of O_2_, some other factors can also affect and sometimes directly limit the catalytic activity of FIH-1, such as Fe2^+^, 2-OG, physiological imbalances in the nucleotide ratio, oxidative stress, and TCA cycle intermediate accumulation in OXPHOS cells. All these factors can precipitate the activity of FIH-1 and enhance the transcriptional activity of HIF-1*α*.

Metabolic alterations in cells are reported as one of the hallmarks of cancer. All metabolic reactions of cells are primarily dependent on the normal enzymatic activities mutated in cancer cells. IDHs play a significant physiological function in normal cells, whereas mutated IDHs lose their functions in cancer cells by converting 2-OG to 2-hydroxyglutarate (2-HG). IDH enzymes typically convert isocitrate to 2-OG. IDHs are categorized into two classes of enzymes based on NAD^+^ or NADP^+^. IDH1 and IDH2 depend on NADP^+^/NADPH, whereas IDH3 is dependent on NAD^+^/NADH. IDH1 and IDH2 play crucial roles in various cellular metabolic processes and defend against oxidative stress. IDH1 is present in the cytoplasm, and IDH2 is present in mitochondria; both convert isocitrate to 2-OG and reduce NADP^+^ to NADPH molecules [95]. At this junction, it is appropriate to mention that 2-HG has been recognized as an oncometabolite support for cancer growth. NADPH is further involved in biogenic processes (such as fatty acids and de novo synthesis) and antioxidant mechanisms. It has been reported that these enzymes are mutated in various disease conditions such as cancer: mutated IDH1 and IDH2 lose their normal function in cancer cells.

**Figure 8:**
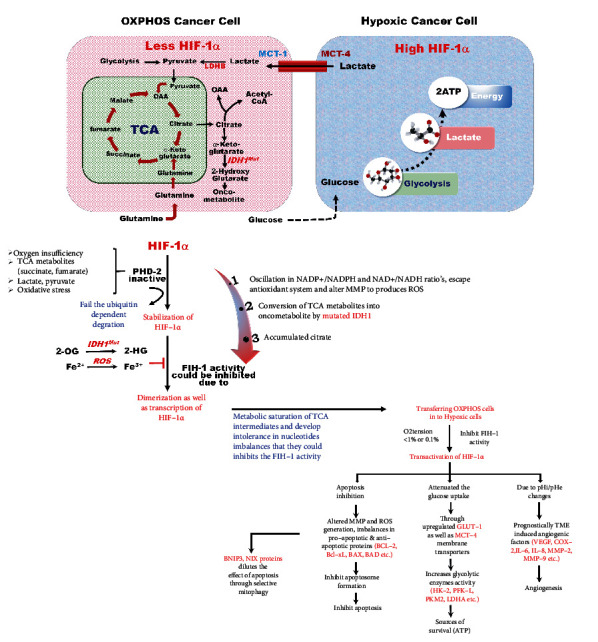
Hypothesized mechanism of FIH-1 activity can regulate the HIF-1*α* transcription in OXPHOS cancer cells and hypoxic cancer cells. This represents the proposed hypothesis based on the regulation of HIF-1*α* transcription between both cancer cells (OXPHOS and hypoxic) by FIH-1, whereas PHD-2 is inactive. PHD-2 regulates proteasomal degradation under normoxia, while it becomes inactivated in OXPHOS cancer cells due to O_2_, TCA intermediates, and oxidative stress by competing for its substrates. After PHD-2 inhibition, HIF-1*α* is stabilized and accumulates in the cytosol and translocates into the nucleus for transactivation. Before transcription, FIH-1 is available to hydroxylate the C-TAD domain of HIF-1*α*. The hydroxylated C-TAD cannot transcriptionally activate HIF-1*α* to prevent the interaction of CBP/p300 coactivator proteins and C-TAD. FIH-1 also depends on O_2_, 2-OG, Fe (II), and ascorbate. FIH-1 has a higher affinity for O_2_ and 2-OG than PHD-2; it continues to work even at ≥1% intracellular O_2_. Apart from O_2_ availability, some other factors may affect and sometimes directly limit the catalytic activity of FIH-1, including the availability of Fe2^+^, 2-OG, and alteration in the nucleotide ratio, due to oxidative stress and citrate accumulation in OXPHOS cancer cells, which can hamper the activity of FIH-1. In normal cells, 2-OG is used by FIH-1 as a substrate for catalytic activity. OXPHOS cancer cells 2-OG are consumed by the mutated IDH1 and converted into 2-HG (FIH-1 inhibitor). Therefore, 2-HG competitively inhibits FIH-1 by occupying the 2-OG catalytic site of this enzyme. In addition, decreased NADPH levels can also affect electron potential and electron transport within the mitochondria, leading to ROS production under mutated IDH1 activity. In addition to PHD-2 inhibition, ROS can also hinder the catalytic activity of FIH-1 through oxidation of its substrate, such as Fe (II) into Fe (III). Lastly, citrate abundantly accumulates in the cytoplasm and can inhibit FIH-1 by itself or the converted form of oxaloacetate (OAA), thereby transferring the OXPHOS cells into complete hypoxic cells. All these factors inhibit FIH-1 catalytic activity, which leads to cancer growth. As a result, modifying FIH-1 activity provides a remarkable strategy for lowering HIF-1*α* transcriptional activity, thereby reducing all of its hypoxic effects and associated consequences.

In normal cells, 2-OG is used by FIH-1 as a substrate for catalytic activity. In OXPHOS cells, 2-OG is utilized by mutated IDH1 to form 2-HG (FIH-1 inhibitor). 2-HG competitively inhibits FIH-1 by occupying the 2-OG catalytic site. In addition, the decreased pool of NADPH can also affect the electron potential and electron transport within the mitochondria, leading to ROS production due to mutated IDH1 activity. In addition to PHD-2 inhibition, ROS can also hinder the catalytic activity of FIH-1 either by oxidizing its substrate (Fe^2+^ into Fe^3+^) or by competitively decreasing its O_2_ selectivity. Lastly, citrate accumulation could be another way to limit the catalytic activity of FIH-1. Furthermore, the concentration of citrate can also increase the imbalance in NADPH, an essential substrate for fatty acid synthesis. Therefore, the citrate can be limited to its conversion rate and utilization in fatty acid synthesis. Subsequently, the citrate abundantly accumulates in the cytoplasm, which could inhibit FIH-1 by itself and transfer the OXPHOS cells into complete hypoxic cells (Figure 8).

From the above, we can hypothesize that the regulation of FIH-1 controls the transcriptional activity of HIF-1*α* through C-TAD, a domain regulated by FIH-1. In normoxia, where cells are fully oxygenated, both PHD-2 and FIH-1 are active and regulate the degradation and transcriptional regulation of HIF-1*α*. A gradual decline in the O_2_ level occurs in the cells located far away from the blood vessels, leading to stabilization of HIF-1*α* due to inactivation of PHD-2. However, FIH-1 remains active and controls the transcriptional activity of HIF-1*α* through C-TAD. As the O_2_ concentration declines further, the transcription of hypoxic genes occurs in a phased manner due to inhibition of both PHD-2 and FIH-1. FIH-1 works as a target to attenuate cancer hypoxia by abrogating HIF-1*α* transcriptional activity through regulating the C-TAD-discerning transcriptional domain. The authors would like to convey the message to readers that HIF-1*α* plays an important role in cancer survival and adaptation to the hypoxic microenvironment via the transcriptional mechanisms, and their transcriptional inhibition could be a promising therapeutic opinion in hypoxia-related malignancies and solid tumors.

## Figures and Tables

**Table 1:** Km and Ki values of the dioxygenase family of enzymes (PHDs and FIH-1).

Enzyme	Km value (*μ*M)			Ki value (*μ*M)		
	O_2_	*α*-Ketoglutarate	Ascorbate	Fumarate	Succinate	Citrate
PHD-1	230	60	170	80 ± 10	350 ± 20	—
PHD-2	250	60	180	60 ± 20	460 ± 70	1800 ± 320
PHD-3	230	55	140	50 ± 15	430 ± 200	180 ± 80
FIH-1	90	25	260	>10000	>10000	110 ± 30

**Table 2:** Specific inhibitors of PHD-2 and FIH-1.

Enzyme	Specific inhibitors	References
PHD-2	IOX4	[96]
	JTZ-951	[97]
FIH-1	N-Oxalyl-D-phenylalanine	[98]

**Table 3:** HIF-1*α* inhibitors under clinical trials.

Treatment agent	Mechanism of action	Status	Study phase	Disease conditions	Intervention/treatment	Clinical trial identifier
RO7070179	HIF-1*α* mRNA antagonist	Completed	Phase I	Hepatocellular carcinoma	Drug: RO7070179	NCT02564614

EZN-2968	Inhibits HIF-1*α* synthesis	Completed	Phase I	Neoplasms Liver metastases	Drug: EZN-2968	NCT01120288

Topotecan	Inhibits HIF-1*α* expression and angiogenesis	Completed	Phase I	Neoplasms	Drug: fluorine-19-fluoroded xyglucose Drug: topotecan	NCT00117013

CRLX101 (nanoformulation of camptothecin)	Inhibits HIF-1*α* expression and angiogenesis	Completed	Phase II	Ovarian cancer Fallopian tube cancer Primary peritoneal cancer	Drug: CRLX101 Drug: bevacizumab	NCT01652079

PT2385	Inhibits HIF-2 dimerization and DNA binding	Completed Active, not recruiting Completed Recruiting Active, not recruiting	Phase II Phase II Phase I Phase I Phase I	Recurrent glioblastoma von Hippel's disease Modifiers of clear cell renal cell carcinoma Healthy Renal cell carcinoma Clear cell renal cell carcinoma Kidney cancer Clear cell renal cell carcinoma Renal cell carcinoma	Drug: HIF-2*α* inhibitor PT2385 Other: pharmacological study Other: laboratory biomarker analysis Other: pharmacogenomic study Drug: PT2385 tablets Drug: PT2385 Drug: [18F] PT2385 Procedure: positron emission tomography/computed tomography Procedure: biopsy Part 1: PT2385 tablets Part 2: PT2385 tablets+nivolumab Part 3: PT2385 tablets+cabozantinib	NCT03216499 NCT03108066 NCT02553356 NCT04989959 NCT02293980

PT2977	Inhibits HIF-2 dimerization and DNA binding	Active, not recruiting Completed Active, not recruiting	Phase II Phase I Phase I	VHL syndrome VHL-associated renal cell carcinoma VHL-associated clear cell renal cell carcinoma Healthy Advanced solid tumors, solid carcinoma, kidney cancer, metastatic renal cell carcinoma, renal cell carcinoma, clear cell renal cell carcinoma, glioblastoma multiforme	Drug: belzutifan Drug: belzutifan Drug: belzutifan	NCT03401788 NCT03445169 NCT02974738

Irinotecan	Inhibits translation of HIF-1*α*	Completed	Phase I	Refractory solid tumors in children	Drug: irinotecan+rapamycin	NCT01282697

PX-478	Inhibits HIF-1*α* protein accumulation and transactivation	Completed	Phase I	Advanced solid tumors Lymphoma	Drug: PX-478	NCT00522652

## Data Availability

Data are available on request.

## Abbreviations

HIF-1: Hypoxia-inducible factor-1
HIF-2: Hypoxia-inducible factor-2
HIF-3: Hypoxia-inducible factor-3
bHLH-PAS: Basic helix-loop-helix/Per-ARNT-Sim
N-TAD: N-terminal transactivation domain
C-TAD: C-terminal transactivation domain
ODDD: Oxygen-dependent degradation domain
HRE: Hypoxia response element
CBP/p300: cAMP response element-binding protein and p300 protein
FIH-1: Factor-inhibiting HIF-1*α*
PHD-2: Prolyl hydroxylase-2
pVHL: von Hippel-Lindau protein
2-OG: 2-Oxoglutarate
2-HG: 2-Hydroxyglutarate
pH_i_: Intracellular pH
pH_e_: Extracellular pH
EGLN1, 2, and 3: Egl nine homolog1, 2, and 3
MMPs: Matrix metalloproteinases
GLUTs: Glucose transporters
HKs: Hexokinases
HK-2: Hexokinase-2
ATP: Adenosine triphosphate
LDHA: Lactate dehydrogenase A
LDHB: Lactate dehydrogenase B
PDK: Pyruvate dehydrogenase kinase
PFK-L: Phosphofructokinase isoform L
GAPDH: Glyceraldehyde 3-phosphate dehydrogenase
PKM2: Pyruvate kinase M2
VEGF: Vascular endothelial growth factor
EPO: Erythropoietin
PGK1: Phosphoglycerate kinase 1
CA 2: Carbonic anhydrase 2
CA 9: Carbonic anhydrase 9
CA 12: Carbonic anhydrase 12
MCT-1: Monocarboxylate transporter-1
MCT-4: Monocarboxylate transporter-4
NHE1: Sodium hydrogen exchanger 1
NAE: Sodium anion exchanger
ETC: Electron transport chain
ROS: Reactive oxygen species
OXPHOS: Oxidative phosphorylation
TME: Tumor microenvironment
NADH: Nicotinamide adenine dinucleotide hydrogen
FAD: Flavin adenine dinucleotide
ADP: Adenosine diphosphate
NBS: Sodium bicarbonate symporter
FASN: Fatty acid synthase
ALA: Alpha linolenic acid
GLA: Gamma linolenic acid
SREBPs: Sterol regulatory element-binding proteins
HDACs: Histone deacetylases
ECM: Extracellular matrix
EMT: Epithelial-to-mesenchymal transition
BBAP-1: 4-[7-(Acetyloxy)-2-ethyl-2H-chromen-3-yl] phenyl acetate
NADPH: Nicotinamide adenine dinucleotide phosphate hydrogen
GSH: Glutathione
NF-*κ*B: Nuclear factor kappa-light-chain-enhancer of activated B cells
PCNA: Proliferating cell nuclear antigen
OMM: Outer mitochondrial membrane
IMM: Inner mitochondrial membrane
FUNDC1: FUN-14 domain-containing protein 1
PINK-1: Phosphatase and tensin homologue- (PTEN-) induced putative kinase
BNIP3: B cell lymphoma 2 interacting protein 3
BNIP3L: BNIP3-like protein
NIX: NIP3-like protein X
LC3: Light chain 3
LIR: LC3-interacting region
CK2: Casein kinase 2
Src: Tyrosine-protein kinase Src
Drp1: Dynamin-related protein 1
OPA1: Optic atrophy 1
OAA: Oxaloacetate
MMP: Mitochondrial membrane potential
Bcl-2: B cell lymphoma 2
Bcl-xL: B cell lymphoma 2-extra large
BAX: Bcl-2-associated X protein
BAD: Bcl-2-associated agonist of cell death
IL-6: Interleukin-6
IL-8: Interleukin-8
Beclin 1: BECN1
LC3B: Light chain 3B
ER: Endoplasmic reticulum
IMS: Intermembrane space
MARCH5: Membrane-associated ring finger (C3HC4) 5
Fis1: Fission 1
Mfn1 and 2: Mitofusins 1 and 2
PGM: Phosphoglycerate mutase
IDHs: Isocitrate dehydrogenases
IDH1*^MUT^*: Mutated isocitrate dehydrogenase 1
PGAM5: Phosphoglycerate mutase family member 5
uPAR: Urokinase plasminogen activator
PPP: Pentose phosphate pathway.

## Supplementary Materials

Supplementary Materials Supplementary Materials. This figure represents the signaling of HIF-1*α* in different conditions. In normoxia, HIF-1*α* is regulated by PHD-2 and FIH-1. PHD-2 is responsible for the hydroxylation of two proline residues (P-402 and P-564) of ODDD at N-TAD, and in parallel, FIH-1 hydroxylates one asparagine (Asn-803) residue of C-TAD under normal conditions of O_2_, 2-OG, ascorbate, and Fe (II). The hydroxylated region of N-TAD is recognized by the pVHL protein, a tumor suppressor protein that attaches to them for proteasomal destruction by ubiquitination via E3- ubiquitin ligase activation. Hydroxylated C-TAD, on the other hand, prevents CBP/p300 proteins from interacting, reducing the HIF-1*α* transcriptional program. When the O_2_ availability is low (<21% O_2_, intermittent hypoxia), PHD-2 becomes inactive, leading to the stabilization of HIF-1*α*. FIH-1, on the other hand, governs the discerning characteristic in low oxygen tension (≥1% O_2_) and regulates the transcriptional function of HIF-1*α*. Under hypoxia (<1% O_2_), HIF-1*α* is fully stabilized and further processed for the transcriptional program after binding with CBP/p300 proteins because FIH-1 inactivates, and it is not able to interrupt the C-TAD and CBP/p300 complex. This complex subsequently enters the nucleus, dimerizes with its companion subunit HIF-1*β*, and binds to the HRE protein, expressing hypoxic genes that promote cancer progression. All in all, FIH-1 could be a potential target for controlling transcription signaling in hypoxia-induced cancers.
