# Supplementary figures and images for: Regulation of Transactivation at C-TAD Domain of HIF-1α by Factor-Inhibiting HIF-1α (FIH-1): A Potential Target for Therapeutic Intervention in Cancer
Source: Oxid Med Cell Longev. 2022 May 10;2022:2407223. doi: 10.1155/2022/2407223 (PMC9113874; doi:10.1155/2022/2407223)

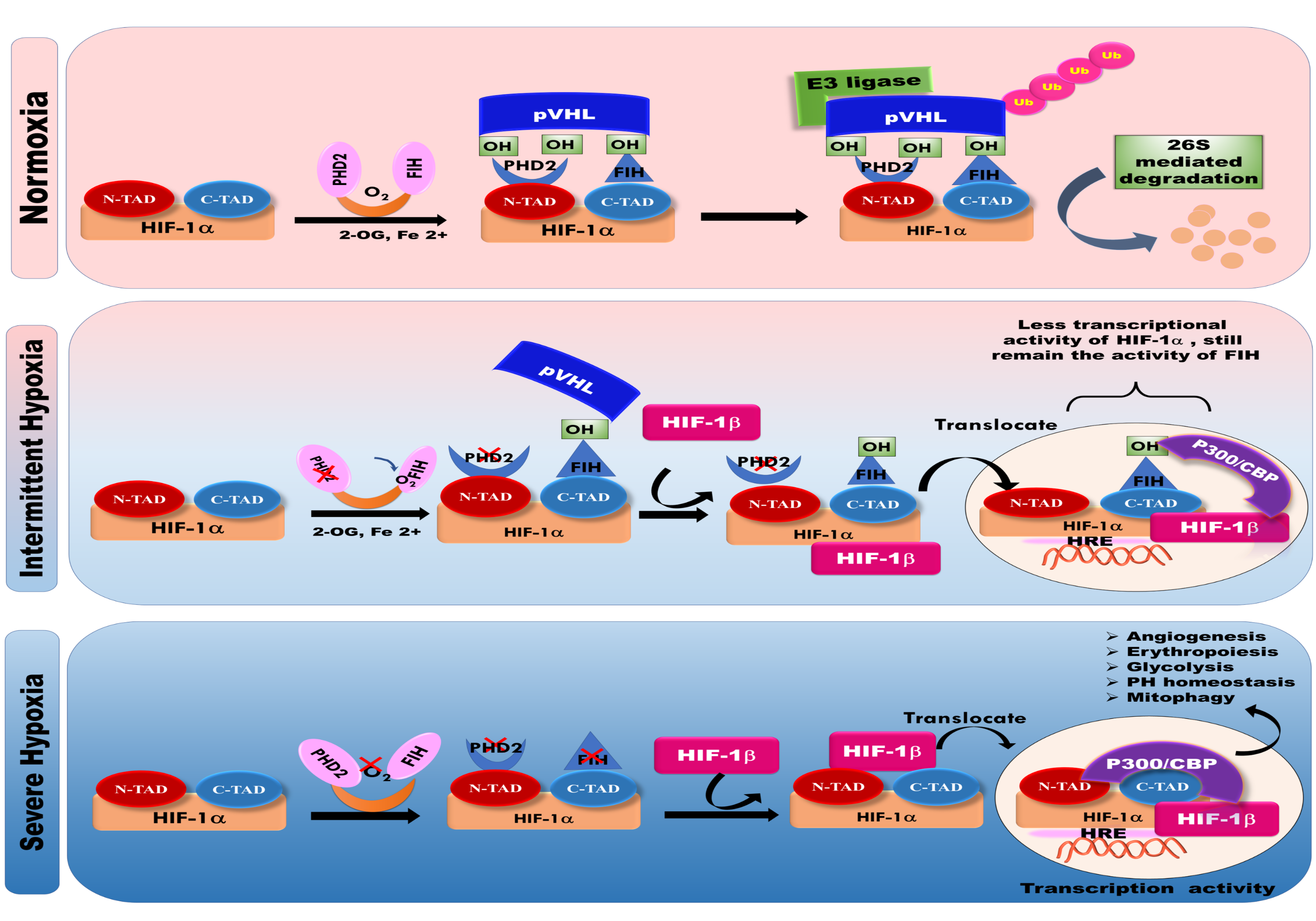

Supplement: Supplementary Materials — This figure represents the signaling of HIF-1α in different conditions. In normoxia, HIF-1α is regulated by PHD-2 and FIH-1. PHD-2 is responsible for the hydroxylation of two proline residues (P-402 and P-564) of ODDD at N-TAD, and in parallel, FIH-1 hydroxylates one asparagine (Asn-803) residue of C-TAD under normal conditions of O2, 2-OG, ascorbate, and Fe (II). The hydroxylated region of N-TAD is recognized by the pVHL protein, a tumor suppressor protein that attaches to them for proteasomal destruction by ubiquitination via E3- ubiquitin ligase activation. Hydroxylated C-TAD, on the other hand, prevents CBP/p300 proteins from interacting, reducing the HIF-1α transcriptional program. When the O2 availability is low (<21% O2, intermittent hypoxia), PHD-2 becomes inactive, leading to the stabilization of HIF-1α. FIH-1, on the other hand, governs the discerning characteristic in low oxygen tension (≥1% O2) and regulates the transcriptional function of HIF-1α. Under hypoxia (<1% O2), HIF-1α is fully stabilized and further processed for the transcriptional program after binding with CBP/p300 proteins because FIH-1 inactivates, and it is not able to interrupt the C-TAD and CBP/p300 complex. This complex subsequently enters the nucleus, dimerizes with its companion subunit HIF-1β, and binds to the HRE protein, expressing hypoxic genes that promote cancer progression. All in all, FIH-1 could be a potential target for controlling transcription signaling in hypoxia-induced cancers. [file 2407223.f1.png]
